# Dietary intake, physical activity and sedentary behavior and association with BMI during the transition to parenthood: a prospective dyadic study

**DOI:** 10.3389/fpubh.2023.1092843

**Published:** 2023-06-02

**Authors:** Vickà Versele, Lara Stas, Dirk Aerenhouts, Tom Deliens, Christophe Matthys, Leonardo Gucciardo, Roland Devlieger, Peter Clarys, Annick Bogaerts

**Affiliations:** ^1^Department of Movement and Sport Sciences, Faculty of Physical Education and Physiotherapy, Vrije Universiteit Brussel, Brussels, Belgium; ^2^Department of Development and Regeneration, Faculty of Medicine, KU Leuven, Leuven, Belgium; ^3^Support for Quantitative and Qualitative Research (SQUARE), Core Facility of the Vrije Universiteit Brussel, Brussels, Belgium; ^4^Department of Biostatistics and Medical Informatics, Vrije Universiteit Brussel, Brussels, Belgium; ^5^Clinical and Experimental Endocrinology, Department of Chronic Diseases and Metabolism, KU Leuven, Leuven, Belgium; ^6^Department of Endocrinology, University Hospitals Leuven, Leuven, Belgium; ^7^Department of Obstetrics and Prenatal Medicine, Faculty of Medicine, Vrije Universiteit Brussel, Brussels, Belgium; ^8^Obstetrics and Prenatal Medicine, University Hospital Brussel, Brussels, Belgium; ^9^Obstetrics and Gynaecology, University Hospital KU Leuven, Leuven, Belgium; ^10^Department of Obstetrics, Gynaecology and Fertility, Wilrijk, Belgium; ^11^Faculty of Medicine and Health Sciences, Centre for Research and Innovation in Care (CRIC), University of Antwerp, Antwerp, Belgium; ^12^Faculty of Health, University of Plymouth, Plymouth, United Kingdom

**Keywords:** body mass index, nutrition, physical activity, sedentary behavior, pregnancy, postpartum, mother, father

## Abstract

**Introduction:**

Little is known on how diet, physical activity (PA) and sedentary behavior (SB) changes during pregnancy and after childbirth in primiparous couples. Moreover, it is unclear how potential behavioral changes are associated with changes in BMI. This study examined changes in diet, PA and SB, and their association with changes in BMI in couples transitioning to parenthood.

**Methods:**

Dietary intake (FFQ), PA, SB (both Actigraph GT3X accelerometers) and BMI of women and men were assessed at 12 weeks of gestation, 6 weeks and 6 months postpartum. Data were analyzed using dyadic longitudinal data analyses techniques.

**Results:**

In women, a decrease in fruit intake, an increase in alcohol intake, an increase of light-intensity PA, and a decrease in SB were observed from the beginning of pregnancy up to 6 months postpartum. Decreases in fruit intake between 6 weeks and 6 months postpartum was associated with increases in BMI. Men did not show significant dietary changes, while an increase in light-intensity PA and a decrease in moderate-to-vigorous PA (MVPA) was observed at 6 months postpartum when compared to 12 weeks of gestation. Paternal increases in “avoidance food group” intake were associated with increases in BMI between baseline and 6 weeks postpartum. No associations of changes in BMI and changes in PA and SB were found.

**Discussion:**

Not only mothers but also fathers experienced unfavorable changes in lifestyle during the transition to parenthood, with impact on BMI changes. This highlights the need to monitor unhealthy changes in lifestyle and body weight in both parents when expecting a child and after childbirth.

**Clinical trial registration:**

Clinicaltrials.gov, NCT03454958.

## 1. Introduction

Healthy dietary patterns, engaging in regular physical activity (PA) and limiting sedentary behavior (SB) during pregnancy are beneficial for the health outcomes of both mother (e.g., decreased risk for the development of gestational diabetes) and offspring (e.g., decreased risk for preterm birth) (1–4). In the postpartum period, healthy dietary behavior and adequate PA are important to help women lose the (excessive) gestational weight gained during pregnancy and prevent postpartum weight retention (PPWR) (5, 6). Indeed, stable energy balance related behavior (EBRB), i.e., when energy intake (i.e., dietary intake) equals energy expenditure (i.e., PA and SB), is important to control weight related outcomes. As the transition to parenthood is a period during which both parents are at risk for unfavorable changes in body weight, knowledge is needed on how changes in body weight or body composition [for which BMI can be used as a proxy] are associated with changes in EBRB.

Indeed, changes in EBRB during the transition to parenthood have been described in previous research (7, 8). However, literature about changes in dietary intake and PA and SB levels during pregnancy and postpartum is conflicting, and moreover, there is little research on how the transition to parenthood impacts fathers(-to-be) (9). Moreover, both parents can influence each other’s behavior and therefore, studies should take into account that couple members’ data is correlated (10). Yet, such a dyadic research approach, where it is possible to investigate women and men’s data simultaneously, has only been scarcely used (10, 11).

Finally, most research focused on changes in BMI and EBRB without looking into associations between changes in BMI and changes in EBRB (9). This knowledge is needed for both mothers-(to-be) and fathers-(to-be) in order to intervene and prevent an unbalanced EBRB and (long-term) weight gain or retention during the pregnancy and postpartum period. Moreover, research showed that there is an urgent need for couple-focused research and interventions (12). Therefore, the primary aim of this study was to investigate to what extent EBRB in fathers and mothers changes from early pregnancy to 6 weeks and 6 months postpartum. Secondly, the aim was to investigate how changes in BMI can be explained by changes in EBRB within the couples.

## 2. Methods

### 2.1. Study design and participants

A multi-center observational follow-up study (TRANSPARENTS study; Trial registration: Clinicaltrials.gov, NCT03454958) with a focus on changes in body weight, body composition and EBRB during the transition to parenthood was set up. For more details on the study protocol and methodology, the interested reader is referred to the earlier published study protocol (13). In short, sample size calculations indicated that a sample of 124 couples (i.e., 248 participants) would be needed to study the main outcomes (i.e., changes in body weight, body composition and EBRB) of the TRANSPARENTS study (13). We aimed for geographical distribution of our sample by recruiting participants for the quantitative follow-up study were recruited from two regions (Flemish Region (including two provinces: “Vlaams Brabant” and “Limburg”) and Brussels Capital Region). Eventually, 152 expectant nulliparous heterosexual couples were recruited between June and December 2018 from four participating hospitals in Belgium, of which 144 could be used for further analysis (14). During their first prenatal visit (gestational week 8–10 weeks), eligible couples (≥18 years old, singleton nulliparous pregnancy) were asked to participate, after which baseline measurements were performed at the end of the first trimester (PG12: 12 weeks of gestation ±2 weeks). Follow-up measurements were carried out at 6 weeks (PP6WK), 6 months (PP6M) and 1 year (PP12M) postpartum. Due to the lock-down and measures imposed in the context of the fight against the Coronavirus Disease 2019 (COVID-19) it was impossible to measure all couples during the last measurement moment (PP12M), as described in the original protocol (13), and PP12M could thus not be included in the analysis. Measurements were performed in the hospitals or at the participants’ homes, depending on their preference. The study was approved by the Medical Ethics Committee of the University Hospital of Vrije Universiteit Brussel (Brussels, Belgium) (B.U.N. 143201835875) and all participants signed a written informed consent before the start of the study.

### 2.2. Measurements

#### 2.2.1. Body mass index (BMI)

During each study visit (PG12 – PP6M), anthropometric measurements were performed and participants received an accelerometer which they had to wear for seven consecutive days, starting the day after the study visit. An online questionnaire was sent to the participants 1 week following the study visit (day 8 after each study visit), and a reminder to complete the questionnaire was sent three times with intervals of 1 week.

#### 2.2.2. Energy balance related behavior (EBRB)

Physical activity (PA) and sedentary behavior (SB) were measured using accelerometers (GT3X+, Actigraph, United States), worn on the right hip for seven consecutive days for at least 12 h/day. Couples were instructed to wear the accelerometers in the week following the anthropometric measurements and to keep a daily log to provide information on activities during which the accelerometer was removed (e.g., water activities). Daily logs were used for manual data-cleaning and to assess if registered non-wear time (≥60 min of consecutive zeros) was indeed non-wear time or sedentary time, making use of Actilife Software. Days during which accelerometers were worn for <10 h were removed, and participants with less than 5 days of valid accelerometer data were excluded from the analysis (15). Time spent in light intensity PA (LIPA) and moderate-to-vigorous intensity PA (MVPA) were calculated using Freedson’s cut points and were presented in minutes per day. SB was defined with a cut-off of 99 counts per minute and presented as percentage of daily accelerometer wear time.

One week after the study visit, an online questionnaire to assess dietary intake, socio-demographic characteristics and child feeding practices (the latter only for PP6WK – PP6M) was sent to the participants. Dietary intake was assessed over the last 30 days using a validated 22-items Food Frequency Questionnaire (FFQ), of which questioned food groups were based on the national Food Based Dietary Guidelines (16). For each food group, average intake per month was questioned (never, 1–3 days/months, 1 day/week, 2–4 days/week, 5–6 days/week, every day), after which the average portion per day had to be chosen from a list of predefined portions specific for each food group. Average daily intakes of fruits (g/day) (i.e., fresh, canned, frozen and dried fruit), vegetables (g/day) (i.e., raw and cooked vegetables and soups), alcohol (ml/day), “avoidance food group” (g/day) (i.e., sugary drinks, sweet and salty snacks, sauces, sweet spreads and processed meat products), and total calorie intake (kcal/day) were calculated.

Socio-demographics (including parental age, educational level and family-household income) were assessed at baseline (PG12) and weeks of exclusive breastfeeding were questioned at follow-up (PP6WK – PP6M). Sex and gestational age of the new-born and data of the last recorded weight of the mother before delivery were retrieved from the medical records. The latter was needed to calculate gestational weight gain (GWG) (kg), which was done by subtracting the last recorded body weight before delivery with body weight measured at PG12.

### 2.3. Statistical analysis

All data were analyzed using R version 4.1.2 (17) and RStudio (18), and IBM SPSS Statistics version 28.0.0.0 (19). *p*-values < 0.05 were considered statistically significant. For data-cleaning and exploration the R packages dplyr, tidyverse, and ggplot2 were used (20–22). Descriptive statistics (means and standard deviation (SD)) and descriptive groupwise comparisons (independent samples t-tests) were calculated by making use of the R package tableone (23).

Missingness mechanisms were inspected for women and men separately both at each time point and longitudinally. Missingness not at random (MNAR) had to be excluded for using the multilevel models. For this purpose, data patterns were tested using R package mice and margin plots were created using the R package VIM (24, 25). Missingness analysis showed MNAR for data collected at PP12M, with missing values up to 51% for some of the outcome variables. This was another argument not to include PP12M and focus on the first three time points (PG12 – PP6M) for data analyses. Margin and pattern plots for PG12, PP6WK and PP6M were checked and showed no indication for MNAR. Possible clustering effects of the four participating hospitals for the main variables (BMI, fruit, vegetables, alcohol, “avoidance food group,” LIPA, MVPA, SB) were checked by means of data visualizations and residual intraclass correlations (ICC), showing that these were negligible. As a result, we did not control for recruitment center in the final models. Missing data were imputed as five multiple datasets by making use of the “Impute Missing Data Values”-function in SPSS, as described in the IBM Manual on missing data (26). Constraints were added as minimum and maximum values for each imputed value from the minima and maxima of the original data. All outcomes presented in this paper represent the analysis from the pooled results, except for the sample characteristics and model diagnostics (i.e., the -2-Restricted Log Likelihood (-2LL) and AIC/BIC values), for which the results from the original dataset were used as the pooled results are not shown in SPSS.

For the first and primary aim, investigating changes in EBRB, the data were analyzed using a multilevel model for each EBRB variable (i.e., different models for dietary intake and PA/SB variables) for longitudinal dyadic data that treats the three levels in the data (repeated measures nested within persons nested within couples) as two levels of random variation (27). This because, in case a three level model would be considered (repeated measures nested within persons nested within couples), it is not possible to have random variability at level two and the model would be saturated at the middle level (27). Therefore, the conceptual three-level model is typically represented by a two-level multilevel model (28). In particular, level one contains the variation due to within-person repeated measures for fathers and mothers (i.e., EBRB variables, covariates and time) and level two contains the between-couple variability across female and male partners (i.e., sex) (29, 30). A two intercept approach was used to study changes in EBRB over the three time points for women and men. That is, the general intercept is removed and two dummy variables for sex are included, together with interaction effects between each predictor variable and these dummies. This approach allows to explicitly estimate the effects of women and men separately by specifying a separate intercept for both women and men, allowing subgroup analyses (27). Note that level one, therefore, represents a two-equation multivariate system (one equation for fathers and one for mothers) which are linked because each time point of the fathers’ equation has a corresponding time point in the mothers’ equation, and the father’s and mother’s residuals are allowed to correlate at any given time point (27). Level two represents between-couples differences. The multilevel model is specified as a marginal model with an unstructured correlations covariance structure (UNR in SPSS), allowing different residual variances for women and men and showing the ICC between men and women in the output. The Restricted Maximum Likelihood (REML) approach was used as an estimation procedure.

For the second aim, investigating how changes in BMI are associated by changes in EBRB over time, difference scores for the outcome (i.e., BMI) and predictor variables (i.e., fruit, vegetables, alcohol, “avoidance food group,” total energy intake, LIPA, MVPA, SB) variables were calculated by subtracting variables measured at PP6WK with those at PG12 and variables measured at PP6M with those at PP6WK, after which continuous predictor variables were grand mean centered. Covariates which were considered (i.e., household family income, gestational age at birth, GWG and weeks of exclusive breastfeeding) were also grand mean centered. Data were imputed based on grand mean centered values. As difference scores could not be calculated for participants of which only the baseline measurement was available, only a total of 133 out of 144 couples were included in the analysis. Two models were built, one to investigate the association between changes in BMI and changes in dietary intake (which we will refer to as the diet model), and one to investigate the association between changes in BMI and changes in PA and SB (which we will refer to as the PA&SB model). Similar models were used as described above, i.e., two-level two-intercept unstructured models estimated with REML. The models were built using a stepwise approach, during each step, two-way interaction terms between sex (i.e., mother and father) and time (i.e., difference between PP6WK and PG12, and difference between PP6M and PP6WK), two-way interaction terms between sex and the EBRB predictors or covariates (i.e., sex*variable), as well as three-way interaction terms between sex, time and EBRB predictors (i.e., sex*time*EBRB predictors) were added. In a first step, level one variables (i.e., covariates at the individual level: age, educational level and the EBRB predictors) were added. Secondly, two-way covariate interactions with *p* > 0.10 where removed, and EBRB predictors with both two- and three-way interactions with p > 0.10 were removed. In a third step, level two variables (i.e., covariates at the dyadic level: household family income, gestational age, breastfeeding and GWG) were added. Finally, two-way covariate interactions with *p* > 0.10 were removed from the models.

## 3. Results

### 3.1. Sample characteristics

Of the initial 152 couples measured at baseline, 144 were eligible to be included in the analyses. Participants who underwent a miscarriage (*n* = 2 couples), with IVF pregnancy, as this might already have affected women’s body weight irrespective of the pregnancy and postpartum period itself (31, 32) (*n* = 3 couples) or with a history of bariatric surgery in the mother (*n* = 3 couples) were excluded together with their partner from the analyses. A total of 15 women and 17 men were lost-to follow-up because they could not be contacted again or did not want to participate in the follow-up measurements (lost-to follow-up between PG12 – PP6WK: 12 women and 13 men; between PP6WK – PP6M: 3 women and 4 men). Sample characteristics for women and men at baseline are shown in Table 1.

**Table 1:** Sample characteristics.

	Couples (*n* = 144)		
	Mother (*n* = 144)	Father (*n* = 144)	*p*-value
**Hospital (*n* (%))**			
UZ Leuven	67 (46.5)		
ZOL	16 (11.1)		
UZ Brussel	25 (17.4)		
Jessa Hospital	36 (25.0)		
Baseline measurement in weeks of gestation (mean (SD))	12.8 (1.0)		
First follow-up measurement in weeks postpartum (mean (SD))	6.4 (1.0)		
Second follow-up measurement in weeks postpartum (mean (SD))	26.3 (1.0)		
Sex of the child: girls (*n* (%))	78 (54.2)		
Gestational age in weeks (mean (SD))	39.2 (1.6)		
**Household income *n* (%)**			
Less than 2,000 €/month	5 (3.5)		
2,000–3,000 €/month	9 (6.3)		
3,000–4,000 €/month	63 (44.4)		
4,000–5,000 €/month	43 (30.3)		
More than 5,000 €/month	22 (15.5)		
**Level of education *n* (%)**			<0.001
Primary education	1 (0.7)	6 (4.3)	
Secondary education	12 (8.6)	39 (27.9)	
College	61 (43.9)	34 (24.3)	
University	65 (46.8)	61 (43.6)	
Age in years (mean (SD))	29.3 (3.4)	31.2 (4.0)	<0.001
Gestational weight gain in kg (mean (SD))	13.4 (4.6)	N.A.	
Exclusive breastfeeding in weeks (mean (SD))	11.2 (8.6)	N.A.	
BMI at 12 weeks of gestation in kg/m^2^ (mean (SD))	24.3 (4.8)	25.4 (3.9)	0.034
BMI at 6 weeks postpartum in kg/m^2^ (mean (SD))	25.2 (4.8)	25.8 (4.1)	0.254
BMI at 6 months postpartum in kg/m^2^ (mean (SD))	24.7 (5.1)	25.8 (3.98)	0.053

### 3.2. Changes in EBRB during the transition to parenthood

Result for this first and primary aim are shown in Tables 2, 3. Women significantly decreased their fruit intake at 6 weeks and 6 months postpartum compared to baseline (PP6WK – PG12 (SE): −38.6 g/day (14.1), *p* = 0.006; PP6M – PG12 (SE): −60.9 g/day (14.0), *p* < 0.001), and significantly increased their alcohol intake from baseline to postpartum (PP6WK – PG12 (SE): +12.3 g/day (3.8), *p* = 0.001; PP6M – PG12 (SE): +21.0 g/day (3.7), *p* < 0.001). A trend towards a significant increase for total energy intake was found at 6 weeks postpartum compared to baseline (PP6WK – PG12 (SE): +69.9 kcal/day (40.2), *p* = 0.083). No significant changes were found for women’s changes in vegetables group intake and “avoidance food group” intake, nor for fruit, vegetables, “avoidance food group” and total energy intake for men during the transition to parenthood (all *p* > 0.05). A trend towards a significant decrease was found for alcohol intake at 6 months postpartum compared to baseline (PP6M – PG12 (SE): −19.2 g/day (10.2), *p* = 0.062).

**Table 2:** Changes in dietary intake during the transition to parenthood.

	MODEL fruit (g/day)		MODEL vegetables (g/day)		MODEL alcohol (g/day)		MODEL “avoidance food group” (g/day)		MODEL total energy intake (kcal/day)	
Predictors	Estimates (SE)	*p*-value	Estimates (SE)	*p*-value	Estimates (SE)	*p*-value	Estimates (SE)	*p*-value	Estimates (SE)	*p*-value
**Fixed part**										
Mother										
Intercept	206.3 (9.8)		228.8 (10.1)		6.4 (2.6)		272.0 (17.7)		1322.6 (28.1)	
12 weeks of gestation (reference)										
6 weeks postpartum	−38.6 (14.1)	**0.006**	−16.2 (14.6)	0.268	12.3 (3.8)	**0.001**	13.8 (26.3)	0.601	69.9 (40.2)	0.083
6 months postpartum	−60.9 (14.0)	**<0.001**	−6.6 (14.7)	0.656	21.0 (3.7)	**<0.001**	−20.8 (24.3)	0.393	−32.8 (39.5)	0.406
Father										
Intercept	103.7 (8.5)		211.1 (10.6)		107.8 (7.1)		360.3 (31)		1502.3 (37.1)	
12 weeks of gestation (reference)										
6 weeks postpartum	6.8 (12.3)	0.582	8.8 (15.4)	0.568	−13.5 (10.2)	0.184	53.3 (43.8)	0.224	23 (55.1)	0.677
6 months postpartum	5.4 (12.2)	0.660	0.1 (15.1)	0.994	−19.2 (10.3)	0.062	16.9 (43.9)	0.700	−73.3 (57.2)	0.202
**Random part**										
Estimate of variance mother	13470.5 (925.5)		14594.7 (1032.1)		943.5 (78.5)		39072.6 (3641.6)		106998.5 (7551.5)	
Estimate of variance father	10374.3 (708.3)		15304.4 (1071.8)		7203.0 (497.9)		136995.3 (9662.3)		195765.7 (13953.0)	
ICC mother—father	0.18 (0.05)		0.22 (0.05)		0.28 (0.05)		0.27 (0.05)		0.09 (0.05)	
**Deviance**										
−2LL	9578.084		9772.834		8350.366		10919.731		11512.171	
*N* (couples)	144		144		144		144		144	
Observations without imputation	407		407		407		407		407	
Observations with imputation	432		432		432		432		432	

ICC, residual intraclass correlation; −2LL, −2-Restricted Log Likelihood. Bold values are *p*-values considered statistically significant.

**Table 3:** Changes in PA and SB during the transition to parenthood.

	MODEL LIPA (min/day)		MODEL MVPA (min/day)		MODEL SB (%/day)	
Predictors	Estimates (SE)	*p*-value	Estimates (SE)	*p*-value	Estimates (SE)	*p*-value
**Fixed part**						
Mother						
Intercept	248.7 (5.0)		27.1 (1.4)		67.5 (0.6)	
12 weeks of gestation (reference)						
6 weeks postpartum	15.6 (7.2)	**0.030**	−4.5 (2.0)	**0.021**	−2.5 (0.9)	**0.004**
6 months postpartum	51.8 (7.3)	**<0.001**	−2.4 (2.0)	0.226	−5.4 (0.9)	**<0.001**
Father						
Intercept	273.5 (6.4)		41.3 (1.9)		64.2 (0.8)	
12 weeks of gestation (reference)						
6 weeks postpartum	14.0 (9.2)	0.128	−4.0 (2.6)	0.121	−1.2 (1.2)	0.285
6 months postpartum	23.1 (9.1)	**0.011**	−5.4 (2.7)	**0.047**	−1.8 (1.1)	0.123
**Random part**						
Estimate of variance mother	3475.4 (253.6)		259.8 (19.3)		54.3 (4.2)	
Estimate of variance father	5670.4 (415.4)		481.6 (33.1)		93.0 (6.8)	
ICC mother—father	0.16 (0.05)		0.22 (0.05)		0.18 (0.05)	
**Deviance**						
−2LL	8552.999		6629.052		5357.908	
*N* (couples)	144		144		144	
Observations without imputation	400		400		400	
Observations with imputation	432		432		432	

LIPA, light intensity physical activity; MVPA, moderate-to-vigorous intensity physical activity; SB, sedentary behavior; ICC, residual intraclass correlation; −2LL, −2-Restricted Log Likelihood. Bold values are *p*-values considered statistically significant.

In terms of PA and SB, both women and men experienced significant changes at 6 weeks and 6 months postpartum compared with the beginning of pregnancy. Women significantly increased LIPA-levels (PP6WK – PG12 (SE): +15.6 min/day (7.2), *p* = 0.030; PP6M – PG12 (SE): +51.8 min/day (7.3), *p* < 0.001). Maternal MVPA levels at 6 weeks postpartum significantly decreased (PP6WK – PG12 (SE): −4.5 min/day (2.0), *p* = 0.021) compared to baseline, but this effect disappeared at 6 months postpartum (PP6M – PG12 (SE): −2.4 (2.0), *p* = 0.226). Also women’s SB decreased (PP6WK – PG12 (SE): −2.5%/day (0.9), *p* = 0.004; PP6M – PG12 (SE): −5.4%/day (0.9), *p* < 0.001). For men, a significant increase in LIPA was found at 6 months postpartum compared to baseline (PP6M – PG12 (SE): +23.1 min/day (9.1), *p* = 0.011), while a significant decrease was found for MVPA (PP6M – PG12 (SE): −5.4 min/day (2.7), *p* = 0.047). No significant changes in men’s SB were found. Results from the different models on changes in dietary intake are shown in Table 2, and results from the different models to investigate changes in PA and SB are shown in Table 3. Raw data of changes in EBRB can be found in Supplementary Tables S1, S2. Figures of changes in dietary intake, PA and SB can be found in Supplementary Figures S1–S8.

The ICC for all dietary intake, PA and SB variables are small but positive. For example, fruit intake of men and women within couples is positively correlated (*r* = 0.18).

### 3.3. Association between changes in BMI and changes in EBRB during the transition to parenthood

Difference scores of the outcome (i.e., BMI) and predictor EBRB variables can be found in Supplementary Tables S3–S5. The final diet model contained two-way interactions with sex for time, household family income, GWG, fruit group and the “avoidance food group” and three-way interactions with sex and time for fruit group and the “avoidance food group.” The vegetables group, alcohol and total energy intake were non-significant predictors and thus removed from the models. Likewise for the non-significant covariates (educational level, gestational age at birth and weeks of exclusive breastfeeding). For the PA&SB model, no significant predictors could be included after inclusion of the level one predictors, and thus no model was built.

Results of the diet model can be found in Table 4. For women, a negative association between changes in BMI and changes in fruit intake was found between 6 weeks and 6 months postpartum, i.e., a decrease of the average daily intake of fruit with 100 g between PP6WK and PP6M corresponded to an increase in BMI of 0.24 kg/m^2^ (*p* = 0.031) in that period. Moreover, changes in BMI (PG12 – PP6WK/PP6M) could be explained by associations with some of the investigated covariates (i.e., household family income – negative association, and GWG – positive association). Each €1000/month higher household family income corresponded with an average decrease in BMI of 0.18 kg/m^2^ (*p* = 0.029), and for each additional kg of GWG there was an average increase in BMI of 0.04 kg/m^2^ (*p* = 0.009).

**Table 4:** Association of changes in BMI with changes in dietary intake.

	Changes in BMI between 12 weeks of gestation and 6 weeks postpartum		Changes in BMI between 6 weeks and 6 months postpartum		Time-independent variables	
Predictors	Estimates (SE)	*p*-value	Estimates (SE)	*p*-value	Estimates (SE)	*p*-value
**Fixed part**						
Mother						
Intercept	1.13 (0.11)		−0.33 (0.11)			
Household family income (€1000/month)					−0.18 (0.08)	**0.029**
GWG (kg)					0.04 (0.02)	**0.009**
Fruit group intake (100 g/day)	0.01 (0.09)	0.908	−0.24 (0.11)	**0.031**		
“Avoidance food group” intake (100 g/day)	−0.12 (0.07)	0.101	0.08 (0.07)	0.275		
Father						
Intercept	0.38 (0.07)		0 (0.07)			
Household family income (€1000/month)					0.01 (0.06)	0.900
Fruit group intake (100 g/day)	−0.02 (0.08)	0.773	−0.15 (0.09)	0.083		
“Avoidance food group” intake (100 g/day)	0.08 (0.03)	**0.029**	−0.05 (0.04)	0.224		
**Random part**						
Estimate of covariance mother	0.61 (0.05)		0.61 (0.05)			
Estimate of covariance father	1.56 (0.14)		1.56 (0.14)			
ICC mother—father	0.24 (0.06)		0.24 (0.06)			
**Deviance**						
−2LL	1483.990		1483.990			
*N* (couples)	133		133			
Observations without imputation	259		259			
Observations with imputation	266		266			

ICC, residual intraclass correlation; −2LL, −2-Restricted Log Likelihood. Bold values are *p*-values considered statistically significant.

After excluding from the model all non-significant predictor variables and interactions, an exact prediction of changes in BMI can be made. Women’s changes in BMI between 12 weeks of gestation and 6 weeks postpartum (BMI_womanPG12PP6WK_), and between 6 weeks postpartum and 6 months postpartum (BMI_womanPP6WKPP6M_) can be predicted as follows:

BMI_womanPG12PP6WK_ = 1.11–0.20*household family income (1,000€/month) + 0.04*GWG (kg).

*BMI*_*woman*PP6WKPP6M_ = −0.36–0.19*household family income (1,000€/month) + 0.05*GWG (kg) – 0.25*fruit group intake (100 g/day).

For men, a negative trend toward significance was found for the paternal association between changes in BMI and changes in fruit group intake (−0.15 kg/m^2^ (0.04), *p* = 0.083). A significant positive association was found between changes in BMI and changes in “avoidance food group” intake between 12 weeks of pregnancy and 6 weeks postpartum; an increase of average daily “avoidance food group” with 100 g in that period corresponded to an increase in BMI of 0.08 kg/m^2^ (*p* = 0.029).

Men’s changes in BMI between 12 weeks of gestation and six weeks postpartum (BMI_manPG12PP6WK_) can be predicted as follows:

BMI_manPG12PP6WK_ = 0.37 + 0.08* “avoidance food group” intake (100 g/day).

## 4. Discussion

This study aimed to investigate how EBRB changes in both members of a couple during the transition to parenthood, and how changes in BMI were associated with changes in EBRB. Our findings showed that mothers decreased their fruit intake from the beginning of pregnancy up to 6 months postpartum while increasing their alcohol intake. No changes in vegetables, “avoidance food group” and total energy intake were found. Moreover, mothers increased their LIPA over the study period. MVPA decreased at 6 weeks postpartum compared to the beginning of pregnancy, but restored to the initial level at 6 months postpartum. Finally, also maternal SB levels decreased across the transition to parenthood. For fathers, no average changes in dietary intake were observed. Paternal LIPA increased at 6 months postpartum compared to the beginning of pregnancy, while MVPA decreased. Paternal SB did not change significantly over the period studied. Furthermore, changes in maternal BMI were positively associated with GWG and negatively associated with household family income and changes in daily fruit intake. Paternal changes in BMI were positively associated with daily changes in “avoidance food group” intake. No associations between changes in BMI and changes in PA and SB were found for mothers, nor for fathers.

Only limited studies are available studying changes in EBRB during the transition to parenthood, while most studies investigated changes during pregnancy, or studied differences between mothers and non-mothers (9, 33–35). Our findings concerning changes in dietary intake during this period are somewhat conflicting with the literature. In particular, we found that maternal fruit intake decreased, while vegetables intake remained stable, whereas most studies found that fruit and vegetable intake generally improved during and after pregnancy (35–40). One other longitudinal study found that fruit intake in first-time mothers decreased compared to non-mothers (33). However, a common finding among all these studies, which is also confirmed by Belgian food consumption data, is that intake of healthy food groups such as fruits and vegetables for most women appear to be below the recommended daily intake (33, 35–41). The pregnant women from our sample had a fruit intake of 206 g/day which is indeed below the recommended 250 g/day. Moreover, this intake is higher in comparison with Belgian women in the same age group of 18–39 years old (i.e., 101 g/day) (41), but lower compared to intakes in pregnant women in other countries (42, 43). Also for the other food groups, baseline values of vegetables intake were for example on average higher compared to the average of Belgian adults in this age group, whereas alcohol and “avoidance food group” intake was lower compared to the average intakes in the Belgian population (41, 44, 45). However, one has to take into account that dietary intake was questioned using a FFQ. Underestimations is a common limitation when using FFQs (46). Different dietary assessment methods used in diet studies therefore makes comparing absolute intake values between studies difficult. The dietary intake data in combination with the SES characteristics of our sample nevertheless might suggest that our sample is a rather health-conscious sample when compared to the general population, a common limitation to studies investigating health behavior (47). The observed decrease in fruit intake with 60.9 g/day (i.e., a decrease of 29.5% of baseline intake) at 6 months postpartum is a worrisome trend. Secondary data analysis in the context of this discussion showed that change scores in women’s fruit intake ranged from −420 g/day to +420 g/day from the beginning of pregnancy up to 6 months postpartum and changes in vegetable intake ranged from -359 g/day to 420 g/day. Although no average change in vegetable intake was found when considering the entire sample, a subgroup of the sample shows significant decreases or increases in vegetable intake. The downward trend in fruit intake might be even more pronounced in an overall pregnant population, and even more in vulnerable pregnant women (e.g., with a lower SES) (48). National food consumption data from Belgium shows that women with a lower socio-economic status (SES) consume the lowest proportion of healthy food groups such as fruits (49). Women with a low SES moreover seem to experience more barriers for adopting a healthy lifestyle and accumulating more unhealthy habits during pregnancy (48). Decreases in daily fruit intake in women from our sample were associated with increases in maternal BMI. Both should be avoided as adequate fruit intake and preventing excessive weight gain is important for the health of the mother and the growing fetus. It is moreover important to prevent postpartum weight retention, and to limit complications during subsequent pregnancies (50).

For fathers, we did not find any changes in dietary intake during the transition to parenthood. This is in line with studies showing no differences in paternal dietary intake (in terms of fruit, vegetables, soft drinks and fast food) from pregnancy to 1 year after birth (51), but contrasting other findings demonstrating changes in dietary intake (e.g., increased consumption of bread and intake of fibers) and food choice (e.g., more convenient foods) (33). We also found a positive association between changes in “avoidance food group” intake and changes in BMI for fathers between the beginning of pregnancy and 6 weeks postpartum, a period during which fathers are vulnerable for weight gain (14). Previous research showed that fathers(−to-be) experienced changes in their dietary intake in both a healthy (e.g., as result of weight management, health consciousness linked with the pregnancy of their partner) and unhealthy (e.g., cravings, influence of the pregnant partner) direction (7). Paternal changes in “avoidance food group” intake ranged from −587 g/day to +936 g/day between baseline and 6 weeks postpartum (46.4% decreased their “avoidance food group” intake, 52.0% increased their “avoidance food group” intake). This distribution and individual changes in both directions could clarify why no average changes in EBRB intake were found. Despite this, individual slopes might be different when studying associations with changes in BMI (i.e., paternal increase of BMI of 0.4 kg/m^2^ from 12 weeks of gestation to 6 weeks postpartum) might clarifying why a positive association between changes in “avoidance food group” intake and BMI was found during this period. Attention is thus needed for unhealthy changes on an individual basis in paternal diet during the pregnancy period, as this might set the basis for unhealthy habits and body weight on the longer term. Follow-up of fathers’ body weight and lifestyle is not part of standard care yet. Based on these and previous results indicating unhealthy changes in paternal body weight during the transition to parenthood, it is highly recommended to include fathers in pre- and postnatal care trajectories.

Furthermore, as with women, our results reflected sub-optimal intakes of men for essential food groups (e.g., paternal fruit and vegetables intake are below the recommendations of 250 g/day and 300 g/day, respectively) (52), which was also described by others during the pregnancy and postpartum period (42). Additionally, from the EBRB groups studied, our results showed that the highest correlation between diet, PA or SB within the couple was found for the alcohol and “avoidance food group” intake between mother and father (*r* = 0.28 and *r* = 0.27 respectively). Even though these correlations are rather low, they are substantial and should not be statistically neglected when analyzing EBRB in couples. Moreover, concordance of dietary behaviors within couples and the association between partner support and PA behavior has been described (10, 53). This highlights the need to target not only mothers but also fathers in lifestyle interventions during the pregnancy and postpartum period.

As expected, maternal and paternal LIPA increased and maternal SB decreased during the transition to parenthood. This confirms the results from an earlier focus group study during which parents explained that they became more active and less sedentary because of the baby (e.g., walking around or playing with the baby) (8). At 6 weeks postpartum, a decrease in maternal MVPA might be attributed to the recovery after delivery (8). Belgian women start physiotherapy sessions 6 weeks after the delivery which encourages them to start moving again. These sessions and the related support was described as a determinant for changes in postpartum PA behavior (8). It is however difficult to compare the PA and SB results from the present study with results from existing studies. Other longitudinal studies often tend to report domain-specific PA data (e.g., leisure-time PA, household related PA), used self-reported PA, and/or did not include SB data (9, 54). Contrary to our results, showing an increase in LIPA for both parents and a decrease in SB for mothers, some studies described a negative relationship between parenthood and PA and indicated that parents were more inactive compared to non-parents (55). Mothers(-to-be) indeed seem to experience several barriers related to PA during pregnancy and in the early postpartum period, resulting in being less physically active when compared to non-mothers (8, 56). Similarly to our results, other described decreases in MVPA and increases in LIPA, or described a shift in PA (i.e., decrease in leisure-time PA and increase in household related PA). It was found that also fathers spend less time in MVPA compared to childless men, but there is a lack of longitudinal studies to compare our results to (57).

Inadequate diet, lack of engagement in PA or excessive SB during pregnancy are associated with negative health and pregnancy outcomes for both mother and child (1–4, 58, 59). These behaviors are moreover important for weight loss after birth and preventive for the development of mental health problems in the postpartum (6, 60). Women often reduce intake of certain foods (e.g., caffeine rich drinks, alcohol) or decrease engagement in PA because of fears of harming the baby, rather than increasing healthy foods/PA which support a healthy pregnancy and promote the baby’s health (61, 62). Maintaining or obtaining healthy EBRB is likewise important for men due to the association of unhealthy EBRB with increased body weight and non-communicable diseases later in life (63, 64). Furthermore, sub-optimal dietary intakes for women and men during pregnancy are common (42), while our findings show that there is an association between dietary intakes of both parents. Hence, interventions aiming to improve EBRB during the transition to parenthood may benefit from being couple-focused.

### 4.1. Strengths and limitations

A strength of this study was the use of longitudinal dyadic data. Even though studies exist where dietary intake of both partners is investigated, they did not use a statistical dyadic research approach (42). Investigating the couples as a pair instead of independent individuals takes into account the interpersonal interactions and the fact that both parents influence each other which was shown from previous qualitative studies (7, 8, 65). The data of couple members is thus nonindependent. Nonindependence is the phenomenon in which the measurements of two dyad members are intercorrelated (66). When ignoring this nonindependence in the data, it can lead to inaccurate estimates of standard errors, and consequently *p*-values are biased. A dyadic research approach takes statistically into account that two couple members come from the same dyad and allows to investigate women’s and men’s data simultaneously. This is a unique aspect of this research, as existing studies in parents expecting a child have to the best of our knowledge not yet investigated data on a dyadic level. Second, BMI, PA and SB were measured objectively. Objective measurements exclude social desirability bias, and provide more reliable insights into actual levels of PA in a population of pregnant women (67) and expectant fathers (68).

Also some limitations should be considered when interpreting the results. First, we used objectively measured PA but for recorded activities during which accelerometers were removed (from daily logs) data was manually imputed. As this was only for a limited number of activities (between 1.1 and 2.0% depending of sex and measurement moment), we would expect limited bias from this imputation. Moreover, no distinction could be made between different domains of PA. For example, it might be that we would have observed changes in leisure-time PA and/or household-related PA. Secondly, as there was no data collection phase at the end of the pregnancy phase or in the first days postpartum, it cannot be derived whether described changes in EBRB at 6 weeks postpartum when compared to 12 weeks of gestation reflect changes during pregnancy or changes within the first weeks following birth. We would expect that changes are overestimated (i.e., fruit intake probably decreased more early postpartum due to for example the influence of the baby (skipping meals) (7), and LIPA and SB results would probably have been in the other direction if measured by the end of pregnancy due to for example physical restrictions (i.e., growing belly) which makes moving difficult). Third, dietary intake was self-reported, and FFQ derived values for dietary intake of energy, foods and nutrients are prone to underestimations, and thus attention is warranted when comparing dietary intake data with cross-sectional data (46). It is nevertheless considered a reliable tool when used in a longitudinal research design to study pregnancy-related changes in dietary intake (69). A fourth consideration is the use of averages. Group level statistics may not apply to everybody nor do they reflect individual changes, as EBRB might change in different directions (e.g., both increases and decreases in “avoidance food group” intake were described) (7, 8). This might have evened out relevant changes. As a result of averaging individual trajectories, information might get lost and this can lead to a problem of aggregation bias. A possible statistical methodology which can be used to further investigate individual changes not reflected in a group’s mean or average effect, are dyadic latent growth curve (LGC) analyses (70). However, due to non-equal spacing between the measurements, LGC analyses were not possible in this case. Finally, observed changes could even be more pronounced considering that baseline BMI and EBRB might already have changed before the baseline assessment at the end of the first trimester of pregnancy. Ideally pre-pregnancy data would have been used.

## 5. Conclusion

Women decreased their fruit intake during the transition to parenthood, which was associated with increased BMI values between 6 weeks and 6 months postpartum. Men did not show significant changes in dietary intake during the transition to parenthood. However increases in paternal BMI between the beginning of pregnancy and 6 weeks postpartum were found to be associated with increases in “avoidance food group” intake and changes in BMI. For both parents, increases in LIPA from the beginning of pregnancy up to 6 months postpartum were observed, and mothers also decreased their SB during this period. Fathers decreased their MVPA levels from the beginning of pregnancy up to 6 months postpartum. No associations between changes in BMI and changes in PA and SB were found. Not only mothers but also fathers are vulnerable for unhealthy EBRB changes associated with changes in BMI. This has implications on the design and delivery of lifestyle interventions during the pregnancy and postpartum period. Researchers, healthcare providers, or policy makers involved in the development of lifestyle interventions during this critical life phase can use these results to adequately support both parents in order to obtain or maintain a healthy body weight and lifestyle, instead of a focus solely on the mother. This can be done by using a multilevel couple- or family-based approach. Moreover, HCPs could play a crucial role in providing additional support to fathers(-to-be). Recommendations for HCPs are thus needed to facilitate couple-focused interventions.

## Data availability statement

The raw data supporting the conclusions of this article will be made available by the authors, subject to data protection requirements. The anonymized dataset and a data dictionary defining each variable of the dataset can be made available with publication upon reasonable request to the corresponding author. Data will only be shared after approval of the ethical committee that handled the review of the project for which the data would be needed.

## Ethics statement

The studies involving human participants were reviewed and approved by the Medical Ethics Committee of the University Hospital of Vrije Universiteit Brussel (Brussels, Belgium), approved on May 16th 2018 (B.U.N. 143201835875). The patients/participants provided their written informed consent to participate in this study.

## Author contributions

VV, DA, TD, LG, RD, PC, and AB contributed to the conception and design of the study. VV recruited and measured the participants and was responsible for data extraction and wrote the original manuscript. VV and LS performed the statistical analyses. VV, LS, DA, TD, CM, LG, RD, PC, and AB were responsible for reflection and interpretation of the data. LS, DA, TD, CM, LG, RD, PC, and AB revised the manuscript critically. All authors contributed to the article and approved the submitted version.

## Funding

This research was supported by a research grant from The Research Foundation—Flanders (“Fonds voor Wetenschappelijk Onderzoek” (FWO)) with project number G033418 N. RD is holder of an FWO Fundamental Clinical Investigatorship with number 1803311 N.

## Conflict of interest

The authors declare that the research was conducted in the absence of any commercial or financial relationships that could be construed as a potential conflict of interest.

## Publisher’s note

All claims expressed in this article are solely those of the authors and do not necessarily represent those of their affiliated organizations, or those of the publisher, the editors and the reviewers. Any product that may be evaluated in this article, or claim that may be made by its manufacturer, is not guaranteed or endorsed by the publisher.

## Figures and Tables

**Table:** 

−2LL	−2-Restricted Log Likelihood
BMI	Body mass index
COVID-19	Coronavirus Disease 2019
EBRB	Energy balance related behavior
FFQ	Food frequency questionnaire
GWG	Gestational weight gain
ICC	Residual intraclass correlation
LIPA	Light intensity physical activity
MVPA	Moderate-to-vigorous intensity physical activity
MNAR	Missing not at random
PA	Physical activity
PG12	12 weeks of gestation
PP6WK	6 weeks postpartum
PP6M	6 months postpartum
REML	Restricted maximum likelihood
SB	Sedentary behavior
SD	Standard deviation
