# Supplementary material for: Dietary intake, physical activity and sedentary behavior and association with BMI during the transition to parenthood: a prospective dyadic study
Source: Front Public Health. 2023 Jun 2;11:1092843. doi: 10.3389/fpubh.2023.1092843 (PMC10272778; doi:10.3389/fpubh.2023.1092843)
Supplement: Supplementary file 1 [file Data_Sheet_1.docx]

Supplementary Material

# Raw EBRB Data

| **Table S.1:** Raw dietary intake data |  |  |  |  |
| --- | --- | --- | --- | --- |
|  | **Maternal** | | **Paternal** | |
|  | **Mean (SD)** | **Range** | **Mean (SD)** | **Range** |
| Fruit at 12 weeks gestation in g/day | 206.1 (122.7) | 8.0 - 540.0 | 103.4 (102.6) | 0.0 - 424.4 |
| Fruit at 6 weeks postpartum in g/day | 168.1 (120.6) | 8.0 - 540.0 | 106.9 (103.7) | 0.0 - 540.0 |
| Fruit at 6 months postpartum in g/day | 142.6 (110.5) | 0.0 - 540.0 | 104.2 (102.6) | 0.0 - 540.0 |
| Vegetables at 12 weeks gestation in g/day | 228.7 (134.2) | 0.0 - 713.7 | 210.8 (122) | 0.0 - 553.1 |
| Vegetables at 6 weeks postpartum in g/day | 211.8 (110) | 0.0 - 552.5 | 217.7 (129.3) | 20.6 - 713.7 |
| Vegetables at 6 months postpartum in g/day | 221.4 (118.1) | 3.2 - 672.5 | 210.5 (122.4) | 6.9 - 605.6 |
| Alcohol at 12 weeks gestation in g/day | 6.1 (25.4) | 0.0 - 193.1 | 108.3 (93.9) | 0.0 - 450.0 |
| Alcohol at 6 weeks postpartum in g/day | 17.7 (28.8) | 0.0 - 147.4 | 95.2 (81.8) | 0.0 - 353.7 |
| Alcohol at 6 months postpartum in g/day | 26.9 (35) | 0.0 - 193.1 | 89.2 (83.2) | 0.0 - 450.0 |
| Avoidance food group' at 12 weeks gestation in g/day | 262 (171.9) | 8.6 - 763.9 | 357.2 (381.9) | 21.8 - 1675.9 |
| Avoidance food group' at 6 weeks postpartum in g/day | 266.9 (192.3) | 0.0 - 1021.9 | 393.9 (391.4) | 13.7 - 1650.8 |
| Avoidance food group' at 6 months postpartum in g/day | 230.0(171.8) | 0.0 - 892.5 | 356.6 (341.4) | 20.2 - 1611.6 |
| Total energy intake at 12 weeks of gestation in kcal/day | 1317.6 (314.1) | 343.4 - 2092.5 | 1501.9 (467.3) | 286.1 - 2867.3 |
| Total energy intake at 6 weeks postpartum in kcal/day | 1381.6 (336.5) | 590.3 - 2506.6 | 1511.6 (408.5) | 394.3 - 2863.4 |
| Total energy intake at 6 months postpartum in kcal/day | 1280.2 (308.4) | 599.3 - 2180.9 | 1424.9 (452.2) | 567.2 - 2695.6 |

| **Table S.2:** Raw PA and SB data |  |  |  |  |
| --- | --- | --- | --- | --- |
|  | **Maternal** | | **Paternal** | |
|  | **Mean (SD)** | **Range** | **Mean (SD)** | **Range** |
| LIPA at 12 weeks of gestation in min/day | 246.2 (58.7) | 133.2 - 433.6 | 272.8 (77.5) | 120.7 - 480.7 |
| LIPA at 6 weeks postpartum in min/day | 260.0 (51.7) | 151.3 - 411.7 | 285.8 (71.9) | 154.6 - 453.7 |
| LIPA at 6 months postpartum in min/day | 299.2 (59.6) | 180.3 - 502.9 | 296.2 (80.5) | 146.6 - 531.7 |
| MVPA at 12 weeks of gestation in min/day | 26.6 (16.6) | 2.1 - 90.8 | 41.3 (24.9) | 7.4 - 145.3 |
| MVPA at 6 weeks postpartum in min/day | 21.8 (17) | 0.3 - 76.5 | 36.9 (21.4) | 4.7 - 109.2 |
| MVPA at 6 months postpartum in min/day | 23.9 (14.3) | 2.5 - 65.4 | 35.3 (20.7) | 6.0 - 133.3 |
| SB at 12 weeks of gestation in %/day | 67.9 (7.2) | 44.9 - 81.6 | 64.3 (10.4) | 27.3 - 83.9 |
| SB at 6 weeks postpartum in %/day | 65.6 (6.5) | 45.3 - 79 | 63.2 (9) | 40.1 - 80.1 |
| SB at 6 months postpartum in %/day | 62.5 (7.4) | 40.8 - 76.5 | 62.5 (9.7) | 33.1 - 82.1 |

# Difference scores of EBRB data

| **Table S.3:** Differences scores of BMI | | | | |
| --- | --- | --- | --- | --- |
|  | **Maternal** | | **Paternal** | |
|  | **Mean (SD)** | **Range** | **Mean (SD)** | **Range** |
| Changes in BMI (PP6WK - PG12) in kg/m² | 1.1 (1.4) | -2.6 - 5.6 | 0.4 (0.9) | -2.4 - 3.5 |
| Changes in BMI (PP6M – PP6WK) in kg/m² | -0.4 (1.2) | -3.5 - 2.7 | 0.0 (0.7) | -3.3 - 1.5 |

| **Table S.4:** Differences scores of dietary intake data | | | | |
| --- | --- | --- | --- | --- |
|  | **Maternal** | | **Paternal** | |
|  | **Mean (SD)** | **Range** | **Mean (SD)** | **Range** |
| Changes in fruit (PP6WK - PG12) in g/day | -39.6 (113) | -357.8 - 315.0 | -0.1 (83.1) | -327.9 - 315.0 |
| Changes in fruit (PP6M - PP6WK) in g/day | -23.0 (98.5) | -315.0 - 255.0 | -3.2 (81.6) | -315.0 - 315.0 |
| Changes in vegetables (PP6WK - PG12) in g/day | -23.7 (122.4) | -353.1 - 352.1 | -2.1 (109.9) | -286.2 - 426.3 |
| Changes in vegetables (PP6M - PP6WK) in g/day | 8.5 (115.6) | -387.8 - 405.7 | -1.8 (103.5) | -277.9 - 327.1 |
| Changes in alcohol (PP6WK - PG12) in g/day | 11.0 (30.5) | -178.8 - 134.1 | -12.2 (73.0) | -257.0 - 193.1 |
| Changes in alcohol (PP6M - PP6WK) in g/day | 9.0 (32.3) | -121.5 - 178.8 | -10.9 (58.0) | -193.1 - 262.5 |
| Changes in 'avoidance food group' (PP6WK - PG12) in g/day | 15.3 (151.5) | -385.8 - 494.1 | 32.9 (195.8) | -586.5 - 935.7 |
| Changes in 'avoidance food group' (PP6M - PP6WK) in g/day | -29.2 (151.7) | -577.5 - 603.5 | -11.4 (181.4) | -546.7 - 596.1 |

| **Table S.5:** Difference scores of PA and SB data | | | | |
| --- | --- | --- | --- | --- |
|  | **Maternal** | | **Paternal** | |
|  | **Mean (SD)** | **Range** | **Mean (SD)** | **Range** |
| Changes in LIPA (PP6WK - PG12) in min/day | 12.1 (71.1) | -165.6 - 203.1 | 13.6 (50.2) | -86.3 - 165.6 |
| Changes in LIPA (PP6M - PP6WK) in min/day | 37.8 (64.1) | -177.7 - 189.6 | 9.6 (52.4) | -174.9 - 119.3 |
| Changes in MVPA (PP6WK - PG12) in min/day | -5.1 (17.9) | -62.4 - 52.1 | -4.5 (19.8) | -73.8 - 45.1 |
| Changes in MVPA (PP6M - PP6WK) in min/day | 1.7 (17.1) | -57.1 - 45.4 | -1.9 (19.5) | -52.3 - 50.4 |
| Changes in SB (PP6WK - PG12) in %/day | -2.2 (8.2) | -20.2 - 18.9 | -1.4 (6.4) | -21.5 - 15.0 |
| Changes in SB (PP6M - PP6WK) in %/day | -2.9 (7.9) | -25.1 - 18 | -0.5 (6.3) | -13.9 - 19 |

# Figures of changes in EBRB during the transition to parenthood


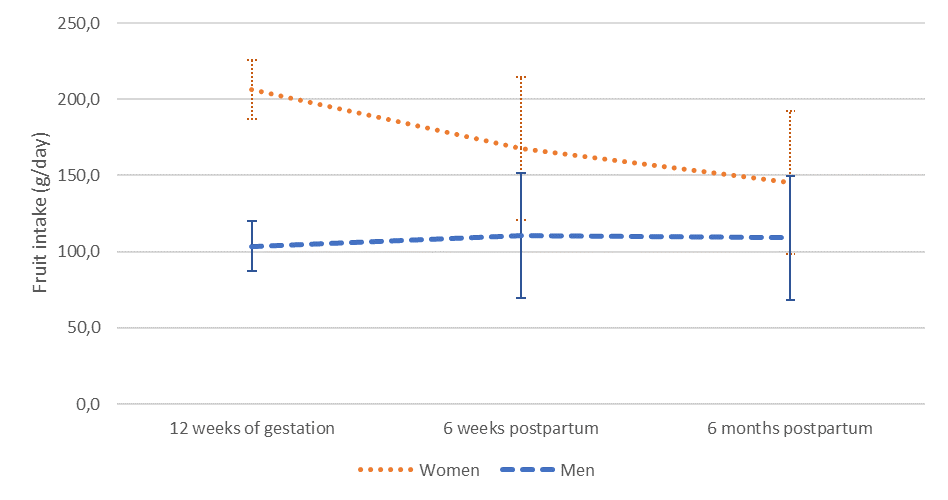


**Figure S.1:** Changes in fruit intake (g/day) during the transition to parenthood.


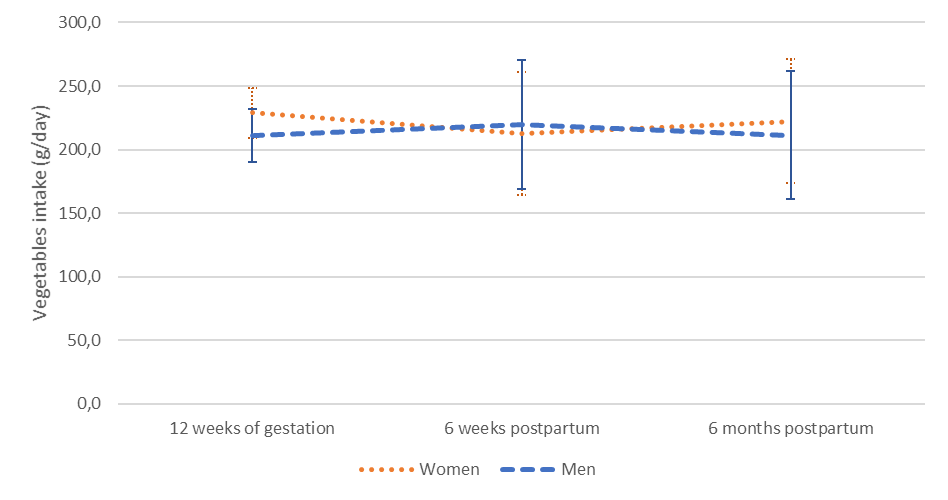


**Figure S.2:** Changes in vegetables intake (g/day) during the transition to parenthood.


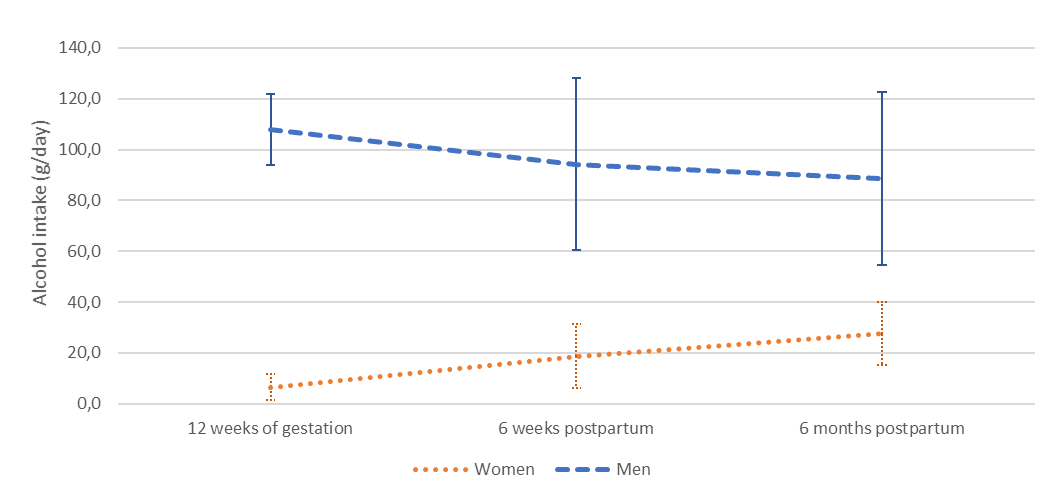


**Figure S.3:** Changes in alcohol intake (g/day) during the transition to parenthood.


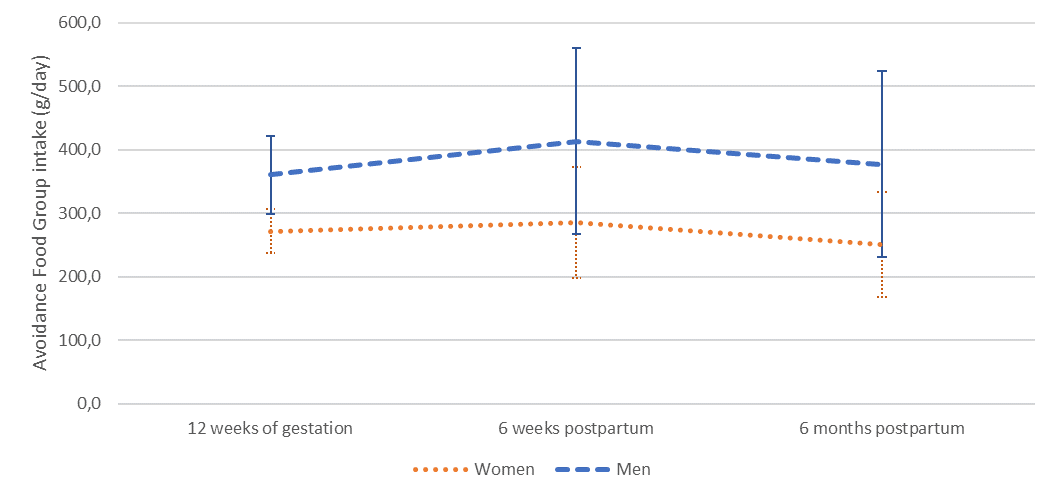


**Figure S.4:** Changes in ‘avoidance food group’ intake (g/day) during the transition to parenthood.


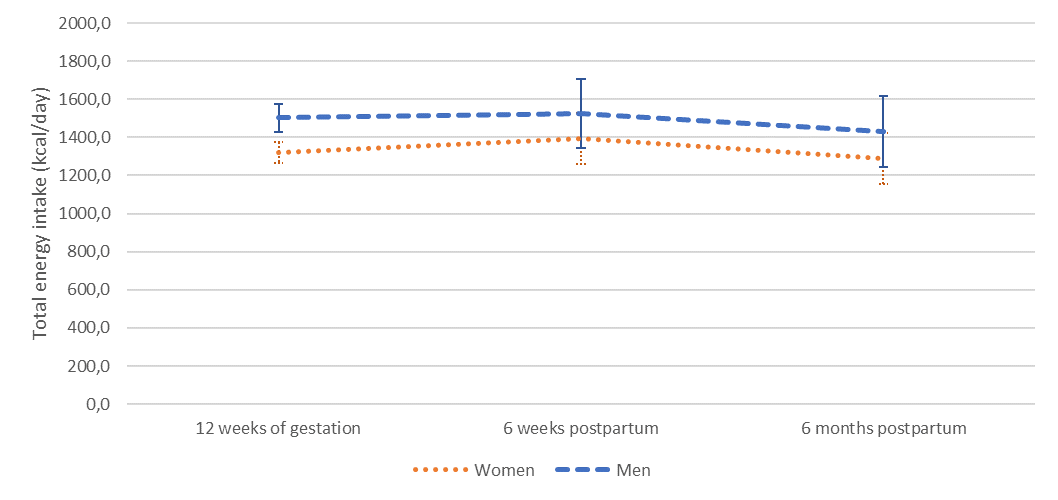


**Figure S.5:** Changes in total energy intake (kcal/day) during the transition to parenthood.


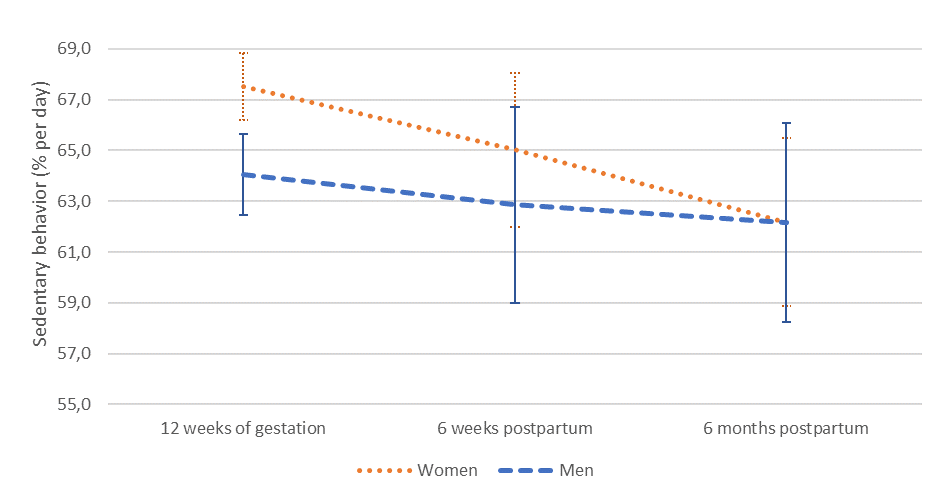


**Figure S.6:** Changes in sedentary behavior (%/day) during the transition to parenthood.

*
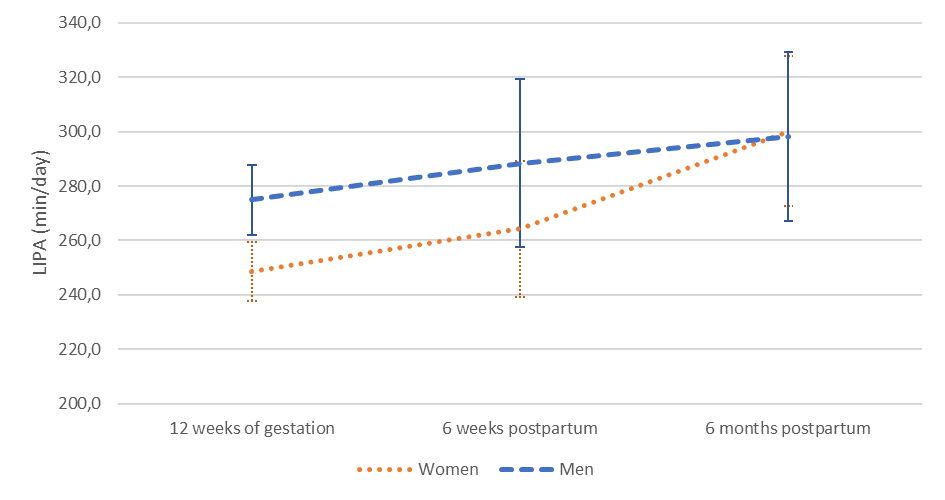
*

**Figure S.7:** Changes in light physical activity (min/day) during the transition to parenthood.

***
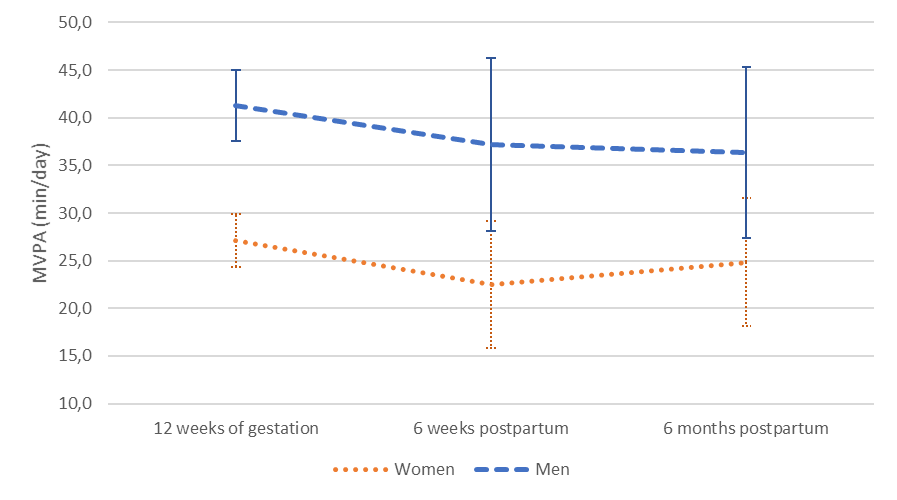
***

**Figure S.8:** Changes in moderate-to-vigorous physical activity (min/day) during the transition to parenthood.
